# Spontaneous Isolated Celiac Artery Dissection: A Case Report

**DOI:** 10.5811/cpcem.2020.5.46906

**Published:** 2020-07-03

**Authors:** Clifford L. Freeman, Aaron J. Lacy, Aubrey Miner, Devin M. Rogers, Austin T. Smith, Karan S. Shah

**Affiliations:** * Vanderbilt University Medical Center, Department of Emergency Medicine, Nashville, Tennessee; † Intermountain Healthcare, Park City Hospital, Department of Emergency Medicine, Park City, Utah; ‡ Indiana University School of Medicine, Department of Emergency Medicine, Indianapolis, Indiana

**Keywords:** SICAD, spontaneous isolated celiac artery dissection, abdominal pain, imaging, computed tomography

## Abstract

**Introduction:**

Abdominal pain is a common chief complaint that can represent a wide breadth of diagnoses, ranging from benign to life-threatening. As our diagnostic tools become more sophisticated, we are able to better identify more causes of potentially life-threatening diseases. One such disease that is relatively unfamiliar to clinicians is spontaneous isolated celiac artery dissection (SICAD).

**Case Report:**

We describe a case of a 46-year-old man who presented to our emergency department with a chief complaint of abdominal pain and was found to have a SICAD and was successfully treated with anticoagulation, antihypertensives, and observation.

**Conclusion:**

It is important for emergency physicians to keep this potentially life-threatening condition in mind and to know the appropriate first steps once identified.

## INTRODUCTION

Abdominal pain is one of the most common presenting chief complaints of patients in the emergency department (ED).^1^ It is a general chief complaint that represents a wide breadth of diagnoses, ranging from benign to life-threatening, and it is critical that emergency physicians be able to differentiate the two. For this reason, abdominal pain can be a challenging chief complaint, as there is often overlap in symptoms and localization of intra-abdominal pathology can be unreliable on physical exam. A combination of labs, imaging, and physical exam is often needed to determine the diagnosis. The management of abdominal pain has changed over time, and recent trends show an increase in computed tomography (CT) being done to aid in the diagnosis.^2^ While increased use of CT carries the risk of radiation exposure, potential of contrast-induced nephropathy, and higher hospital costs, it has led to more reports of diseases that previously could only be identified in the operating room or on autopsy. One such potentially life-threatening diagnosis is spontaneous visceral artery dissection.^3^,^4^ We describe a case of a 46-year-old man who presented to the ED with a chief complaint of abdominal pain and was found to have a spontaneous isolated celiac artery dissection (SICAD) and was successfully treated with anticoagulation, antihypertensives, and observation.

## CASE REPORT

**Image 1:**
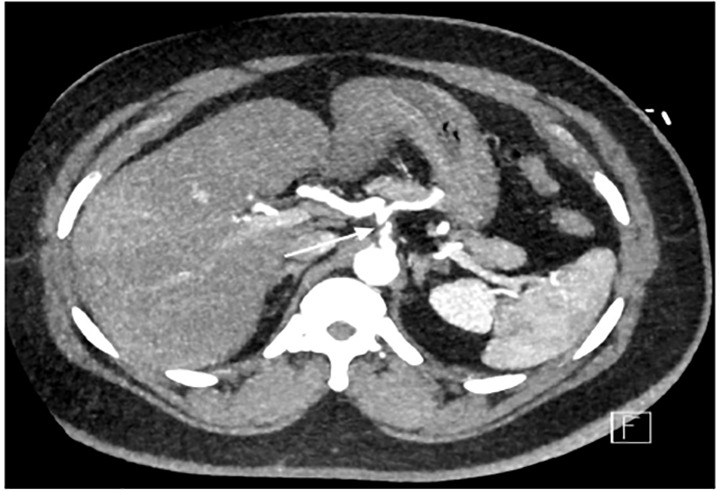
An axial image of a contrast-enhanced computed tomography angiogram showing a dissection flap in the celiac trunk (arrow).

**Image 2:**
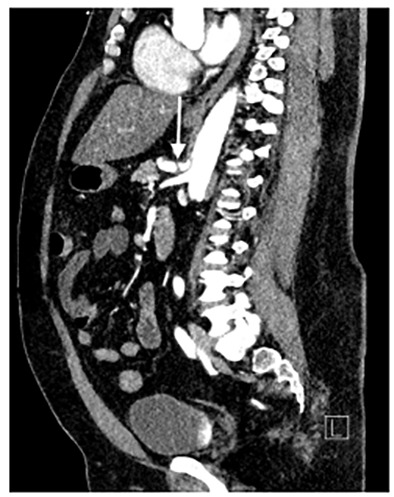
A sagittal image of a contrast-enhanced computed tomography angiogram showing a dissection flap in the celiac trunk (arrow).

**Image 3:**
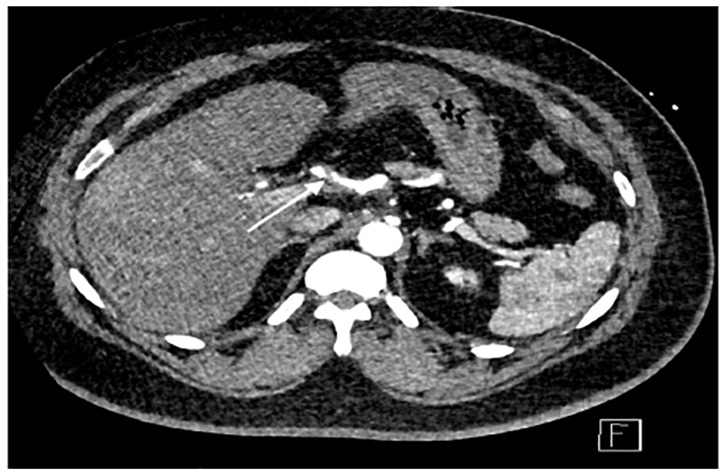
An axial image of a contrast-enhanced computed tomography angiogram showing a dissection flap in the common hepatic artery (arrow).

A 46-year-old male with a past medical history of hypertension and Hodgkin’s lymphoma presented to our ED for evaluation of abdominal pain. He reported that just prior to arrival he had sudden onset pain in his midepigastric region. It was sharp, severe, radiating to his back and was associated with nausea and dyspnea. He was hypertensive with otherwise normal vital signs. On examination he was tender to light palpation in his epigastric region without rebound, guarding, or tenderness elsewhere. He had a normal electrocardiogram without any signs of ischemia. His labs were significant for a white blood cell count of 11.4 × 10^9^ thousands (K) per microliter (mcL) (range 3.6–10.6 K/mcL), with a normal lipase, normal liver function tests, and negative troponin. Given the history and exam, a CT angiogram of the chest, abdomen, and pelvis was ordered, which revealed SICAD (Images 1 and 2) with extension into the common hepatic artery (Image 3).

## DISCUSSION

SICAD is a rare but potentially life-threatening diagnosis.^5^ It is the second leading type of visceral artery dissection after spontaneous isolated superior mesenteric artery dissection.^5^ Visceral artery dissections were first described in 1947 and initially thought to be a fatal injury as all cases reported before 1975 were diagnosed at autopsy.^5^,^6^ However, the implementation of CT angiography has improved the ability to make the diagnosis.^7^ Symptoms can range from asymptomatic incidental findings to severe abdominal pain with bowel ischemia resulting in peritonitis; therefore, the diagnosis requires a high level of clinical suspicion.^7^ The most common profile of patients presenting with SICAD are male smokers with hypertension, although it will also present in those without these comorbidities.^7^ Conservative management is considered the initial treatment for most SICAD patients as long as they do not have bowel ischemia, although there is not a standardized consensus on the best medical therapy.^7^ Most medical treatments performed include a combination of fasting, parenteral nutrition support, pain control, and treatment of hypertension. Two large cohort studies to date have shown no benefit with antithrombotic therapy vs observation in clinical outcomes.^4^,^8^ Our patient was started on an esmolol infusion to control his hypertension along with a heparin infusion at the recommendation of the vascular surgery service. He was admitted and transitioned to oral anticoagulation and antihypertensive medications after his abdominal pain resolved. He did not require intervention and was discharged in good condition several days later.

**CPC-EM Capsule**

What do we already know about this clinical entity?

Spontaneous isolated celiac artery dissection is a rare, but potentially life-threatening diagnosis. There is no consensus on treatment, which ranges from conservative therapy to surgical intervention.

What makes this presentation of disease reportable?

This is a disease process that previously was discovered by autopsy and therefore considered (at that time) to be universally fatal. With the increased utilization of computed tomography imaging, it is being detected more frequently.

What is the major learning point?

This rare, but potentially fatal condition should be considered in the differential diagnosis for abdominal pain; particularly in male smokers with a history of hypertension.

How might this improve emergency medicine practice?

It is important for emergency physicians to consider this potentially fatal diagnosis. Knowledge of this condition, risk factors and presentation will increase the likelihood of detection resulting in life-saving therapies.

## CONCLUSION

Abdominal pain as a chief complaint can vary from benign to catastrophic. Spontaneous isolated celiac artery dissection is relatively rare, and can present from asymptomatic incidental finding to severe pain with bowel ischemia and peritonitis. Early diagnosis is critical to reduce morbidity and mortality and is typically detected on a contrast-enhanced CT. SICAD has a wide presentation range, but often resolves with conservative management. It is important for emergency physicians to keep this potentially life-threatening condition on their differential, and to know the appropriate first steps to take once identified.
